# The association between fear of missing out and mobile phone addiction: a meta-analysis

**DOI:** 10.1186/s40359-023-01376-z

**Published:** 2023-10-17

**Authors:** Yali Zhang, Shijie Shang, Lixian Tian, Lijuan Zhu, Weina Zhang

**Affiliations:** 1 School of Education, Hebei Normal University, Shijiazhuang, China; 2 College of Teacher Education, Hebei Normal University, Shijiazhuang, China; 3 School of Teacher Education, Suqian University, Suqian, China; 4 School of Social Sciences, Universiti Sains Malaysia, Penang, Malaysia

**Keywords:** Fear of missing out, Mobile phone addiction, Meta-analysis

## Abstract

**Background:**

Numerous studies have explored the association between fear of missing out and mobile phone addiction, but there are different viewpoints and the results are inconsistent. This study intends to estimate the strength of the correlation between fear of missing out and mobile phone addiction in general through a meta-analysis, and to analyze the influencing factors of the inconsistent results of previous studies.

**Methods:**

We Searched China National Knowledge Infrastructure Database, Wan fang Database, CQVIP Journal Database、Web of Science Core Collection, Elsevier SD, Springer Online Journals, Medline, EBSCO-ERIC, SAGE Online Journals, PsycINFO, PsycArticles and ProQuest Dissertations and Theses。85 studies (90 independent effect size) were included from 2016 to 2023。The pooled correlation coefficient of the association between fear of missing out and mobile phone addiction was calculated by a random effects model using Comprehensive Meta-Analysis(Version 3.3).

**Results:**

The main effect analysis revealed a high positive correlation between fear of missing out and mobile phone addiction (*r* = 0.47, 95%CI [0.44, 0.50]). Furthermore, the measurements of mobile phone addiction moderated the strength of the association between fear of missing out and mobile phone addiction, with the highest correlation measured using MPATS and the lowest correlation measured using MPDQ. The age, gender, year of publication, cultural background, and the measurements of fear of missing out had no significant effect on the correlation between fear of missing out and mobile phone addiction.

**Conclusion:**

The results indicated that fear of missing out was closely related to mobile phone addiction, which complied with the I-PACE model. Psychological services and mental health services should be developed to reduce the emergence of fear of missing out in the digital age and thus alleviate dependence on devices.

## Background

With the development of Internet technology, mobile phones have become more and more popular around the world. Statistics showed that there are 2.5 billion cellphone users worldwide, accounting for approximately one-third of the world’s population [1]. It is undeniable that powerful cellphone applications enable people to socialize, relax, go shopping and even study online, significantly enriching and facilitating people’s lives. They are designed to elicit the users’ emotional states and gratify their compulsions at any time, which could lead to mobile phone addiction. Mobile phone addiction refers to the behavior of excessive use of various applications carried on mobile phones that leads to the impairment of physical, psychological and social functions [2]. Studies illustrated that it has become increasingly prominent and results in various mental disorders such as anxiety, depression, boredom, sleeping disorders, etc., posing new challenges to regular work schedules and social adaptation [2, 3]. Therefore, this phenomenon has aroused the continuous attention of researchers, and a series of studies have been conducted to explore the causes and formation mechanisms behind it.

From the perspective of I-PACE model, individual physiological factors, cognitive factors, emotional factors and executive function can all have an impact on mobile phone addiction [4]. Recently, researchers have focused on the relationship between fear of missing out and phone dependence, but there are different views. The I-PACE model hold that there is a significant positive correlation between the fear of missing out and mobile phone addiction, while the digital stress model hold that there is a significant negative correlation between them. In addition, the correlation coefficients between the two are also very inconsistent, with reports ranging from 0.16 to 0.80 [5–7]. Therefore, the overall correlation between them has become an urgent problem. This study intends to clarify this by meta-analysis, and analyze the potential factors that may affect the relationship between them, so as to draw more general and accurate conclusions from a macro perspective.

### Inconsistent findings about the association between FoMO and mobile phone addiction

Fear of Missing Out (FoMO) refers to a compound emotional experience characterized by negative feelings, such as fear, anxiety, loss, etc., resulting from the fear of missing out on potential resources or rewarding experiences of someone else [8]. Theoretically, two perspectives exist when discussing the relationship between fear of missing out and mobile phone addiction. One suggested a potential positive correlation between the two variables, and a representative theory is the I-PACE model [4]. It emphasizes that the fear of missing out could result in approach motivation, which motivates individuals to look for digital media to satisfy psychological needs. Cell phones are not only versatile but also easily accessible, so individuals with a higher level of fear of missing out tend to use such media more frequently to satisfy their internal needs and are thus more inclined toward mobile phone addiction [5]. Moreover, the fear of missing out would cause negative psychological distress. Individuals utilize mobile phones for pleasure or purposeless wandering to escape real-life concerns or ease dysphoric moods under avoidance motivation. Eventually, they would get addicted to mobile phones [9]. It also suggested that individuals with a high level of fear of missing out tend to have defects in self-regulation and self-control abilities, which makes it difficult for them to resist the temptation of various applications on mobile phones and then become addicted [8]. Most empirical studies also supported this point of view.

Another perspective suggested that there may be a significant negative correlation between the fear of missing out and mobile phone addiction. From the point of view of digital stress, the fear of missing out has been seen as a kind of digital stress [10]. Though digital stress is triggered by social media use, it could lead to hatred of digital media use. Individuals with a high level of fear of missing out, primarily online, may actively reduce or even avoid using digital media (e.g., cell phones) to alleviate their internal anxiety, which may not lead to cell phone addiction. However, this is only discussed theoretically, and the relationship between fear of missing out and mobile phone addiction has not been adequately explored in studies.

### Potential moderating variables

In the past ten years, numerous studies have explored the association between fear of missing out and mobile phone addiction. However, the results are different. This inconsistency may be attributed to the participant’s age and gender or the measurements used in the research to quantify the variables.

In terms of age, due to the relatively limited range of activities of minors, the main place of activity is school and classroom. The members of the group are familiar with each other, and the activities they carry out and the resources or information they grasp are relatively similar, and the homogeneity phenomenon is relatively high, so the fear of missing is less. Moreover, due to the limited use of mobile phones and other electronic devices, it is difficult for them to make use of such electronic devices for compensation and satisfaction, so it is difficult to be associated with mobile phone addiction. On the contrary, adults have a relatively wide range of activities and communication, and the interaction of heterogeneous group members is easy to make upward social comparisons, thus generating the fear of missing out. Adults are relatively free in using mobile devices, and it is easier to relieve with mobile phones. Thus, the fear of missing out may be more closely related to mobile phone addiction in adults [11, 12].

Gender may also moderate the relationship between fear of missing out and mobile phone addiction. Firstly, as far as the level of psychological resilience is concerned, men have a higher level of psychological resilience than women, especially regarding emotional control. Therefore, when experiencing a high level of fear of missing out, men are more tolerant and tend to be less influenced, so they seldom use mobile phones to talk about their negative feelings and rely less on them, while women are less tolerant and tend to use convenient social media such as mobile phones to relieve their anxiety and rely more on them [7]. Moreover, girls are more socially oriented than boys, while boys are more independent. As a result, girls are more terrified than boys of being excluded from the group and fearing that they will not fit in due to missing resources or opportunities. Therefore, girls experience a higher level of fear of missing out and are more likely to follow others’ network information on the platform through mobile phones than boys [11].

The relationship between fear of missing out and mobile phone addiction may also influenced by cultural background or publication year. As for the cultural background, people from places where they live in collectivism are inclined to develop and maintain excellent interpersonal relationships with others. With an emphasis on integration and harmonious co-existence with the surrounding environment, individuals are more susceptible to the influence of their surroundings and more concerned with others’ acceptance and recognition. Consequently, they are more likely to achieve a higher level of FoMO and find it easier to rely on mobile phones [11, 13]. As for the publication year, with the development of technology, communication technologies today have changed and revolutionized our lifestyles, allowing the mobile phone to become a basic necessity in our daily lives. Individuals with a higher level of FoMO could easily follow up with others using real-time and multiple media applications, resulting in mobile phone addiction among its users [14]. Consequently, the association between FoMO and mobile phone addiction grew stronger and stronger with the increased publication year.

The measurements used in the study could moderate the relationship between FoMO and mobile phone addiction. Firstly, as for the scales of FoMO, the number of questions varies from 1 to 20 [9, 15, 16]. The most widely used scale is FoMO-P, with ten questions [15]. Therefore, the scale with fewer questions may lose some information during the measurement process, causing differences in results. In terms of structures, FoMO-P is a single-dimensional scale measuring the susceptibility to fear of missing out in general contexts. Other questionnaires, such as FoMO-W, have multidimensional structures, measuring the vulnerability to fear of missing out and the fear of missing out in online contexts, which is more comprehensive and may lead to differences in the measurement results [9].

Moreover, as for the scales of mobile phone addiction, the structures are also different, ranging from one to six factors [17–24]. The contents are similar but also different. For example, besides the core contents of mobile phone addiction, SAS-C added two more elements, App Use and App Update [21], which may reflect individuals’ problematic cell phone use more comprehensively, and thus may impact the relationship between fear of missing out and mobile phone addiction.

## Methods

### Literature search and screening

A database search revealed a moderate number of studies examining the fear of missing out. Consequently, the search strategy limited only this variable, so more studies were scanned for further possible articles and included to more fully incorporate the literature on the relationship between fear of missing out and mobile phone addiction. Firstly, the Chinese database(China National Knowledge Infrastructure Journal and Degree Thesis Database, Wan fang Journal, Degree Thesis Database, and CQVIP Journal Database) searched for articles with the keywords “错失恐惧” or “错失焦虑” or “遗漏焦虑”. Secondly, the English database(Web of Science Core Collection, Elsevier SD, Springer Online Journals, Medline, EBSCO-ERIC, SAGE Online Journals, PsycINFO, PsycArticles and ProQuest Dissertations and Theses) was searched with the keywords “Fear of Missing out” or “FoMO”. The search deadline was May first, 2023, and 3,067 articles were retrieved.

**Fig. 1:**
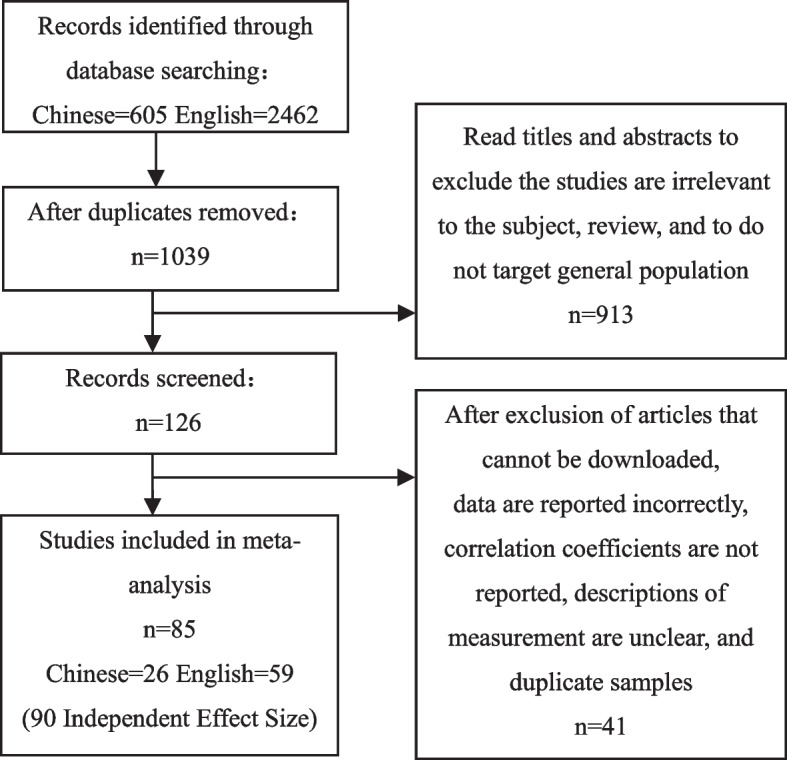
Flow diagram of the search results

Literature was imported using EndNote X9 and screened according to the following criteria:(1) Studies were only included if they reported enough information, including correlation coefficient(r) and sample size, whereas regression coefficients of meta-regression analysis were excluded. (2)There must be an introduction about measurement;(3) For duplicated data, only the more comprehensive ones will be selected. (4) The participants are the general population, and special participants in affected areas during COVID-19 would be excluded. In the end, 85 studies (90 independent effect size) were included from 2016 to 2023. The literature screening process is shown in Fig. 1.

### Coding

Each study was coded according to the following characteristics: first author, year of publication, correlation coefficient, sample size, male ratio, age, and sampling location (Table 1). For the input of the correlation coefficient, if the study did not report the correlation coefficient, but reported the F value, t value, *χ* ^2^ *value, and β value*, the corresponding formula [$r=\sqrt{\frac{{t}^{2}}{{t}^{2}+df}}$ ; $r=\sqrt{\frac{F}{F+{df}_{e}}}$ ; $r=\sqrt{\frac{{\chi }^{2}}{{\chi }^{2}+\text{N}}}$ ; *r* = *β* × 0.98 + 0.05 (*β* ≥ 0); *r* = *β* × 0.98 (*β* < 0)], which is converted to r value before coding(Card, 2012; Peterson & Brown, 2005). Moreover, if the original literature only reported the Pearson correlation between fear of missing out and the sub-dimensions of mobile phone addiction, the formula of ${r}_{xy}=\frac{\sum {r}_{{x}_{i}}{r}_{{y}_{j}}}{\sqrt{n+n(n-1){\stackrel{-}{r}}_{{x}_{i}{x}_{j}}}\sqrt{m+m(m-1){\stackrel{-}{r}}_{{y}_{i}{y}_{j}}}}$ [27] was adopted to calculate the correlation coefficient between fear of missing out and mobile phone addiction for coding. Two evaluators coded independently, and the final consistency was 98%. The difference between the two codes should be corrected by reviewing the original literature and discussing it with the second author. Detailed data is available at the website https://osf.io/qcf67/?view_only=e8a2aabf331244ffa01c19a95d439fe5.

**Table 1:** Basic information of studies included in the meta-analysis

Author	Year	r	Sample size	Male ratio	Counties	Age	Culture	Measurement		Publication	Design	Quality
								FoMO	MPA			
Gökler	2016	0.59	200	0.48	Turkey	adults	37	FoMO-P	Other	Y	C	M
Chotpitayasunondh	2016	0.61	251	0.37	U.K	adults	89	FoMO-P	SAS-SV	Y	C	M
Elhai	2016	0.40	308	0.54	North America	adults		FoMO-P	SAS	Y	C	H
Fuster	2017	0.45	5280	0.24	Latin Country	teenagers		FoMO-P	Other	Y	C	M
Upreti	2018	0.16	75	1.00	India	adults	48	FoMO-P	MPDQ	N	C	M
	2018	0.32	75	1.00	India	adults	48	FoMO-P	MPDQ	N	C	M
	2018	0.19	75	0.00	India	adults	48	FoMO-P	MPDQ	N	C	M
	2018	0.20	75	0.00	India	adults	48	FoMO-P	MPDQ	N	C	M
Gezgin	2018	0.66	161	0.58	Turkey	teenagers	37	FoMO-P	SAS-SV	Y	C	H
Elhai2	2018	0.51	296	0.23	America	adults	91	FoMO-P	SAS	Y	C	M
Blanca	2018	0.27	368	none	Spain	adults	51	FoMO-P	PS	Y	C	M
Franchina	2018	0.20	2663	none	Belgium	teenagers	75	Other	Other	Y	C	H
Sha	2019	0.40	2299	0.61	Germany, Austria, Switzerland	adults		FoMO-P	SAS-SV	Y	C	H
Coskun	2019	0.38	1630	0.45	Turkey	teenagers	37	FoMO-P	Other	Y	C	M
Long	2019	0.38	677	0.59	China	teenagers	20	FoMO-P	SAS-SV	Y	C	H
Santana-Vega	2019	0.53	569	0.39	Spain	teenagers	51	FoMO-P	Other	Y	C	M
Tunc-Aksan	2019	0.42	296	0.54	Turkey	teenagers	37	FoMO-P	SAS	Y	C	M
Schneider	2019	0.31	278	0.26	Germany	adults	67	FoMO-P	Other	Y	C	H
Traş	2019	0.44	608	0.28	Turkey	adults	37	FoMO-P	SAS-SV	Y	C	H
Han	2019	0.62	506	0.41	China	adults	20	FoMO-W	SAS-C	Y	C	M
Ren	2019	0.48	427	0.50	China	adults	20	Other	MPAI	Y	C	H
Chen	2019	0.33	863	0.41	China	adults	20	FoMO-P	SAS-C	N	C	H
Elhai	2020	0.29	1034	0.35	China	adults	20	FoMO-P	SAS-SV	Y	C	M
Elhai	2020	0.40	1097	0.18	China	adults	20	FoMO-P	SAS-SV	Y	C	M
Balta	2020	0.63	423	0.47	Turkey	teenagers	37	FoMO-W	PS	Y	C	M
Liu	2020	0.36	465	0.31	China	adults	20	FoMO-P	SAS-SV	Y	C	M
Coco	2020	0.48	242	0.45	Italy	teenagers	76	FoMO-P	SAS-SV	Y	L	H
Fang	2020	0.29	501	0.29	China	adults	20	FoMO-P	Other	Y	C	H
Handa	2020	0.41	240	0.55	India	adults	48	FoMO-P	Other	Y	C	M
Gugushvili	2020	0.42	426	0.23	Estonia	adults	60	FoMO-P	Other	Y	C	H
Wu	2020	0.69	231	0.00	China	adults	20	FoMO-P	SAS-SV	Y	C	M
Ding	2020	0.45	526	0.48	China	adults	20	FoMO-P	MPATS	Y	C	H
Zhao	2020	0.71	401	0.36	China	adults	20	FoMO-P	MPATS	Y	C	M
Li^a^	2020	0.41	1383	0.53	China	teenagers	20	FoMO-W	Other	N	C	H
Buyukbayraktar	2020	0.50	610	0.47	Turkey	adults	37	FoMO-P	SAS-SV	Y	C	M
Hishan	2020	0.42	296	0.54	Malaysia	teenagers	26	FoMO-P	SAS	Y	C	M
Nosworthy	2020	0.80	252	0.52	America	adults	91	FoMO-P	Other	N	C	M
Tugtekin	2020	0.52	469	0.42	Turkey	adults	37	FoMO-P	Other	Y	C	M
Hu	2020	0.68	689	0.32	China	adults	20	FoMO-S	MPATS	N	C	H
Servidio	2021	0.35	405	0.28	Italy	adults	76	FoMO-P	SAS-SV	Y	C	M
Zhu	2021	0.50	487	0.29	China	adults	20	FoMO-P	Other	Y	C	H
Song	2021	0.51	956	0.00	China	adults	20	FoMO-P	MPATS	Y	C	H
Li^b^	2021	0.42	203	0.34	China	adults	20	FoMO-P	MPAI	Y	C	M
Servidio	2021	0.49	474	0.47	Italy	adults	76	FoMO-P	SAS-SV	Y	C	H
Servidio	2021	0.36	457	0.26	Italy	adults	76	FoMO-P	SAS-SV	Y	C	H
Li^c^	2021	0.45	863	0.41	China	adults	20	FoMO-P	SAS-C	Y	C	H
Li^d^	2021	0.45	1164	0.44	China	adults	20	FoMO-W	MPAI	Y	C	H
Akyol	2021	0.45	235	0.15	Turkey	adults	37	FoMO-P	Other	Y	C	M
Butt	2021	0.75	240	0.50	Pakistan	adults	14	FoMO-P	SAS-SV	Y	C	H
Catiker	2021	0.33	97	0.18	Turkey	adults	37	FoMO-P	SAS	Y	C	M
Li^e^	2021	0.42	927	0.43	China	adults	20	FoMO-P	GSP	Y	C	H
Wu	2021	0.36	851	0.20	China	adults	20	FoMO-P	PS	Y	C	H
Zhang	2021	0.25	318	0.36	China	teenagers	20	FoMO-P	Other	Y	L	H
Cai	2021	0.78	326	0.49	China	adults	20	FoMO-P	MPAI	N	C	M
Cheng	2021	0.49	314	0.32	China	adults	20	Other	PS	Y	C	M
Cui	2021	0.44	924	0.46	China	teenagers	20	FoMO-S	MPAI	N	C	M
	2021	0.41	500	0.50	China	teenagers	20	FoMO-S	MPAI	N	L	M
Fu	2021	0.60	439	0.57	China	adults	20	FoMO-S	PS	Y	C	H
Shen	2021	0.45	825	0.44	China	adults	20	FoMO-P	Other	N	C	H
Xiong	2021	0.64	474	0.10	China	adults	20	FoMO-P	Other	Y	C	M
Zhou	2021	0.79	505	0.51	China	adults	20	FoMO-P	PS	N	C	H
Zhu	2021	0.48	645	0.30	China	adults	20	FoMO-P	Other	N	C	H
Akat	2022	0.52	506	0.29	Turkey	adults	37	FoMO-P	GSP	N	C	M
Alinejad	2022	0.40	447	0.38	Iran	adults	41	FoMO-P	SAS-SV	Y	C	M
Bakioğlu	2022	0.58	419	0.31	Turkey	adults	37	FoMO-P	SAS-SV	Y	C	M
Casale	2022	0.50	364	0.37	Italy	adults	76	FoMO-P	MPAI	Y	C	H
Chi	2022	0.49	938	0.48	China	adults	20	FoMO-P	PS	Y	C	H
Coenen	2022	0.50	807	0.09	Germany	adults	67	FoMO-P	SAS-SV	Y	C	M
Correa-Rojas	2022	0.40	209	0.36	Peru	adults	16	FoMO-P	PS	Y	C	M
García-Castro	2022	0.30	391	0.66	Portugal	adults	27	FoMO-P	PS	Y	C	H
Gong	2022	0.69	975	0.51	China	adults	20	FoMO-P	MPAI	Y	L	M
Kim	2022	0.40	260	0.50	Korea	teenagers	18	Other	Other	Y	C	H
Roberts	2022	0.51	239	0.55	U.S.A	adults	91	FoMO-P	Other	Y	C	H
Sun	2022	0.53	3189	0.63	China	adults	20	FoMO-P	MPAI	Y	C	M
van der Schyff	2022	0.18	275	0.48	U.S.A	adults	91	FoMO-P	PS	Y	C	M
Wu-Ouyang	2022	0.61	777	0.51	China	adults	20	FoMO-P	Other	Y	C	H
Zhao	2022	0.32	501	0.42	China	adults	20	FoMO-P	SAS-SV	Y	C	M
Ding	2022	0.25	515	0.74	China	adults	20	FoMO-P	GSP	Y	C	H
Li	2022	0.25	510	0.51	China	teenagers	20	Other	GSP	N	C	H
Li	2022	0.26	1635	0.35	China	adults	20	FoMO-P	SAS-SV	N	C	H
	2022	0.27	321	0.34	China	adults	20	FoMO-P	SAS-SV	N	L	H
Qin	2022	0.65	1009	0.47	China	adults	20	FoMO-P	GSP	N	C	H
Shi	2022	0.70	709	0.44	China	adults	20	FoMO-W	SAS	Y	C	H
Ye	2022	0.44	6543	0.35	China	adults	20	FoMO-P	MPAI	Y	C	H
Yin	2022	0.44	998	0.42	China	adults	20	FoMO-P	MPAI	Y	C	M
Bajwa	2023	0.02	794	0.47	Pakistan	adults	14	FoMO-P	SAS-SV	Y	C	M
Hudecek	2023	0.24	989	0.26	Germany	adults	67	FoMO-P	Other	Y	C	M
Jin	2023	0.59	517	0.46	China	teenagers	20	FoMO-P	MPAI	Y	C	H
Lv	2023	0.40	357	0.38	China	adults	20	FoMO-W	Other	Y	C	M
Wang	2023	0.44	167	0.57	China	adults	20	FoMO-P	SAS-SV	Y	C	H

Noted: Y represents published, N represents unpublished;

C represents cross-sectional study, L represents longitudinal study;

Culture represents individualism index, obtained from https://www.hofstede-insights.com/

Quality represents literature quality evaluation; H is high quality, M is medium quality

### Literature quality evaluation

We applied the Joanna Briggs Institute Critical Appraisal Checklist for Analytical Cross-sectional Studies to evaluate the methodological quality of original research [28]. There are eight items with four responses, including “Yes, ‘No’, ‘Unclear’ or ' Not applicable. Affirmative responses were assigned 1 point, and the rest of the answers were given 0 points. All the original study were scored between 0 and 8. Each study quality was evaluated based on the score it achieved, where scores < 50% were identified as low quality, 50–80% were identified as medium quality, and > 80% were identified as high quality [25].

### Publication bias control and test

Publication bias refers to the fact that studies with positive results are more likely to be published, so published literature does not fully represent the population of studies done in the field. Unpublished Master’s and Doctoral dissertations were also included in this study, which controlled the impact of publication bias on the results of the meta-analysis to some extent. Additionally, to ensure the reliability of the meta-analysis results, this study used funnel plots and Egger’s regression method to test whether the results whether affected by publication bias. If the graph presented is a reverted funnel shape for funnel plots, the publication bias is too small to influence the results. While for Egger’s regression method, if the linear regression results are non-significant, we could conclude that publication bias doesn’t exist.

### Model selection

Currently, there are two main methods for calculating effect sizes: the fixed-effects model and the random-effects model. The former assumes that the actual effect of different studies is the same and the disparity between the results is caused by random error. The latter believe that the actual effect of various studies may be different and that different outcomes are affected not only by random error but also by different sample characteristics. Since the sampling country and age of the original study may affect the relationship between fear of missing out and mobile phone addiction, this study used a random effect model for estimation. In addition, the heterogeneity test will also be used to determine whether it is necessary to analyze the moderating effect, mainly by looking at the significance of the Q test result and the I^2^ value. If the Q test result is significant or the I^2^ value is higher than 75%, the cause of heterogeneity should be explored as much as possible [26].

### Data processing

This study used the correlation coefficient r as the effect size. Specifically, all the original correlation coefficients r are converted to Fisher-Z values by formula Fisher’$-\mathrm Z\;=0.5\times\text{ln}\left[\frac{1+\text{r}}{1-\text{r}}\right],V_z=1/(n-3)$. Then, the converted effect values are converted to correlation coefficients for interpretation by formula Sumarry *r* = $({e}^{2z}-1)/({e}^{2z}+1)$, Z= Sumarry Fisher’-Z. The main and moderating effects were tested using Comprehensive Meta-Analysis Version 3.3. There are two forms of moderation effect tests. (a) when the moderator is a continuous variable, meta-regression analysis is used to test whether the result is significant; (b) when the moderator is a categorical variable, subgroup analysis is used to examine whether the result is significant.

## Results

### Literature inclusion and quality assessment

This study included 85 papers with 90 Independent Effect Size. The literature is distributed between 2016 and 2023 and covers 16 countries, including 5 longitudinal studies and 80 cross-sectional studies; 71 published and 14 unpublished. all the literature’s quality were above medium level, specifically 42 medium quality and 43 high quality.

### Publication bias test

**Fig. 2:**
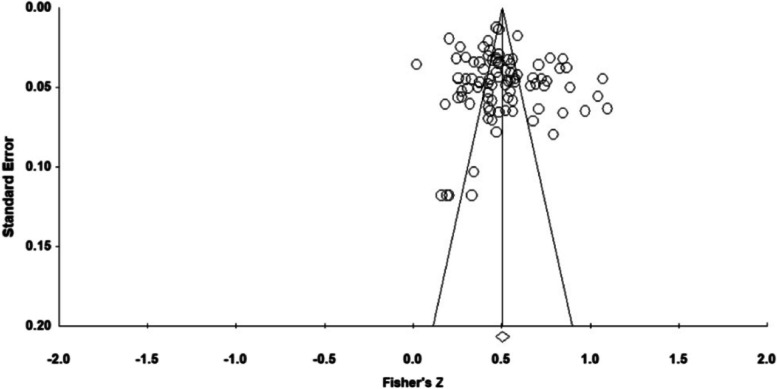
The distribution of effect sizes

Funnel plots (Fig. 2) showed that the effect sizes were concentrated at the top of the graph and distributed evenly on both sides of the overall effect. The Egger’s regression results were insignificant, and the intercept was 1.58, 95% CI [-0.70, 3.85], indicating no significant publication bias in this study. In other words, the results of this meta-analysis are reliable.

### Heterogeneity test

The results showed that the Q value was 2045.30 (*p* < 0.001) and the I^2^ value was 95.65%(>75%), demonstrating that 95.65% of the variance in the effects on the relationship between fear of missing out and mobile phone addiction was due to actual differences in effect size. The results also indicated that the discrepancies between the research results might be interfered by some research characteristic factors, and the moderating effect test can be carried out.

### Main effect test

The random effects model was used to estimate the strength of the correlation between fear of missing out and mobile phone addiction. The results showed that the correlation coefficient between the two variables was 0.47( 95% CI = [0.44, 0.50]). Sensitivity analysis found that after excluding any samples, the effect size: r value fluctuated between 0.46 and 0.47, indicating that the estimated value was relatively stable.

### Moderating effect test

The results of subgroup analysis of categorical variables revealed that the moderating effect of age and measurement of FoMO are insignificant, but the moderating effect of measurement of mobile phone addiction was significant. Specifically, the highest correlation measured using MPATS and the lowest correlation measured using MPDQ (Table 2). The results of meta-regression analysis of continuous variables revealed that the moderating effects of gender and grade are not significant (Table 3).

**Table 2:** Results of subgroup analysis of categorical variables

Moderate variables	*Q*_*B*_	df	p		k	r	95%*CI*	
age	0.91	1	0.34	adults	73	0.47	0.44	0.50
				teenagers	17	0.44	0.36	0.50
Measurement of FoMO	3.59	2	0.17	FoMO-P	75	0.46	0.43	0.49
				foMO-S	4	0.54	0.41	0.65
				foMO-W	6	0.55	0.44	0.64
Measurement of MPA	14.16	7	0.04	SAS-C	3	0.47	0.30	0.62
				GSP	5	0.43	0.29	0.55
				MPAI	12	0.53	0.45	0.60
				MPATS	4	0.60	0.47	0.70
				MPDQ	4	0.22	0.01	0.41
				PS	10	0.48	0.38	0.56
				SAS	6	0.48	0.36	0.59
				SAS-SV	23	0.44	0.38	0.50

**Table 3:** Results of meta-regression analysis of continuous variables

Moderate variables	*b*	95%CI	
Male ratio	0.01	-0.24	0.27
publication year	-0.01	-0.04	0.02
individualism index	-0.001	-0.002	0.002

## Discussion

The relationship between fear of missing out and mobile phone addiction has been the focus of researchers recently. However, the inconsistent results brought difficulties to further research. This study adopted meta-analysis to estimate the correlation coefficient and resolved the disagreement about the effect size of the association. The results demonstrated that the two variables were significantly correlated. Moreover, it was shown that measurements moderated the relationship between fear of missing out and mobile phone addiction, further clarifying why there is such inconsistency with the results of the previous research about the connection between fear of missing out and mobile phone addiction.

### Association between fear of missing out and mobile phone addiction

This study found a high correlation between the fear of missing out and mobile phone addiction, which aligns with the I-PACE model [4], indicating that the fear of missing out was closely related to mobile phone addiction. Because of fear of missing out on others’ rewarding experiences or worrying that others might possess more meaningful experiences than themselves, individuals frequently use their mobile phones to stay connected with others and keep themselves informed of what is going on in the outside world to reduce their inner fears and insecurities [11]. The results revealed that a higher fear of missing out might impair executive function and self-control, making individuals more easily engage in deviant behaviors such as mobile phone addiction [13]. In terms of mental health and digital media use, this suggests that mental health problems make individuals more vulnerable to deviant behavior. Moreover, the previous meta-analysis found that depression(*r* = 0.36), anxiety(*r* = 0.39) and loneliness(*r* = 0.25) were positively correlated with mobile phone addiction [27, 28]. However, the present study showed something different. Compared with depression, anxiety and loneliness, fear of missing out(*r* = 0.47) seemed to have a closer association with mobile phone addiction, suggesting that the relationship between different mental health indicators and mobile phone addiction is diverse. Therefore, it is insufficient to only explore the relationship between general mental health levels and mobile phone addiction.

This study failed to support the view of the Digital Stress Model, which suggests that the emergence of digital stress will reduce the intensity of digital media use and that there should be a negative relationship, but the results of this study suggested a positive relationship. The theory has its rationality, but it needs to be qualified in terms of the conditions and scope of its application. The model emphasizes that digital stress could lead to reducing media use and that digital media use might induce digital stress. Thus, there may be a vicious cycle between digital stress and digital media use [10]. This theory may be suitable to explain the dynamic relationship between fear of missing out and mobile phone addiction in a longitudinal study, i.e., fear of missing out accompanied by novelty would drive individuals to use digital media, which leads to addictive behaviors at the beginning. Later, excessive reliance on digital media accompanied by a sense of fatigue will give rise to digital stress [29]. Of course, some studies have been critical of whether digital stress includes fear of missing out, and the results of this study showed the same. In other words, fear of missing out couldn’t reduce but increase mobile phone addiction because it is not considered a kind of digital stress; stress could reduce the intensity of digital media use [30]. In conclusion, the Digital Stress Model might be appropriate for explaining why digital media use could increase digital stress or subsequent mental health problems, but it cannot explain why mental health problems or digital stress could increase subsequent digital media use.

This study failed to support the view of the Digital Stress Model, which suggests that the emergence of digital stress will reduce the intensity of digital media use and that there should be a negative relationship, but the results of this study suggested a positive relationship. The theory has its rationality, but it needs to be qualified in terms of the conditions and scope of its application. The model emphasizes that digital stress could lead to reducing media use and that digital media use might induce digital stress. Thus, there may be a vicious cycle between digital stress and digital media use [22]. This theory may be suitable to explain the dynamic relationship between fear of missing out and mobile phone addiction in a longitudinal study, i.e., fear of missing out accompanied by novelty would drive individuals to use digital media, which leads to addictive behaviors at the beginning. Later, excessive reliance on digital media accompanied by a sense of fatigue will give rise to digital stress [29]. Of course, some studies have been critical of whether digital stress includes fear of missing out, and the results of this study showed the same. In other words, fear of missing out couldn’t reduce but increase mobile phone addiction because it is not considered a kind of digital stress; stress could reduce the intensity of digital media use [30]. In conclusion, the Digital Stress Model might be appropriate for explaining why digital media use could increase digital stress or subsequent mental health problems, but it cannot explain why mental health problems or digital stress could increase subsequent digital media use.

### Analysis of moderating effects

From the characteristics of the participants, age and gender didn’t moderate the relationship between FoMO and mobile phone addiction. In terms of age, the results are similar in adolescents and adults. This result differs from the previous meta-analysis of the relationship between loneliness and mobile phone addiction, which found that loneliness is more likely to drive adults to mobile phone addiction [28]. It has been illustrated that external restrictions on adolescents’ mobile phone use did not artificially reduce the association between fear of missing out and adolescents’ mobile phone addiction. In addition, the impact of fear of missing out on mobile phone addiction in the era of the Internet may happen regardless of age. In terms of gender, the moderating effect of gender on the relationship between fear of missing out and mobile phone addiction was not significant. This conclusion was consistent with a prior meta-analysis linking smartphone addiction with anxiety and stress [27]. In general, this suggests that the fear of missing out and mobile phone addiction had a cross-gender convergence effect. To put it another way, among various gender groups, mobile phone addiction may be the preferred way of reducing and alleviating the fear of missing out.

Concerning environmental characteristics, neither cultural background nor publication year had a significant moderating effect on the relationship between fear of missing out and mobile phone addiction. In terms of cultural background, this study only selected the index of individualism related to this topic and examined its moderating effect on the relationship between fear of missing out and mobile phone addiction, which was found to be insignificant. The current findings were in line with earlier meta-analyses on the connection between smartphone dependence and anxiety and stress, in which no evidence of cultural differences was found, indicating that there may be a cross-cultural convergence effect on the relationship between fear of missing out and smartphone addiction [27]. This phenomenon may be related to the gradual popularization of cellphone use around the world. As digital natives, mobile phones have been integrated into people’s daily lives and have become essential tools for individuals to be connected. They are indispensable tools for learning about other people’s development, accessing important resources and keeping an eye on changes around them, which could reduce the fear of missing out immediately in some way. So there is no cultural difference in the relationship between fear of missing out and mobile phone addiction [12, 14]. In terms of the year of publication, this study found it insignificant with the connection between fear of missing out and mobile phone addiction, which was different from previous studies. In previous research, the year of publication could positively moderate the relationship between mobile phone use, depression, anxiety and stress. The more recently published the literature, the stronger the relevance is [31]. This may be because the literature included in this study was written after 2016, and the survey’s participants were primarily from Generation Z, which may hardly reflect generational differences. In summary, the effect of publication year on the relationship between FoMO and mobile phone addiction was not significant, and further exploration is needed in the future.

From the measurement characteristics, this study revealed that instruments of mobile phone addiction not fear of missing out could significantly moderate the relationship between fear of missing out and mobile phone addiction. Regarding the measurement of fear of missing out, this study involves three measurement tools: FoMO-P, FoMO-W, and FoMO-S [9, 15, 16]. Although the structure and number of questions are different, these three instruments may essentially convergent, and FoMO-W and FoMO-S are mostly enriched with the foundation of FoMO-P. For example, FoMO-W is an adapted version of FoMO-P. Though some new items have been added to this scale, FoMO-P is still the main topic among the 12 items, and 7 of which have been retained, so they are similarly related to cell phone addiction. In terms of measurements of mobile phone addiction, five scales were used, with MPATS and MPDQ yielding the highest and lowest correlations, respectively. The discrepancy may be related to the difference in purpose and basis of scale development. The former may pay more attention to the tendency of addiction, while the latter is a diagnostic tool for mobile phone addiction disorder based on the DSM diagnostic criteria. Although both scales reflect mobile phone addiction to some extent, the different levels of addiction and targeted symptoms may have contributed to the differences in measurement results. Consequently, the factors that cause the inconsistency mainly come from the chosen instruments rather than some demographic and environmental factors. According to the findings, we should concentrate on preventing and minimizing the onset of the fear of missing out, which could be a new strategy to reduce mobile phone addiction in the future.

### Implication and limitations

This study is the first to systematically sort out the relationship between fear of missing out and cell phone addiction using a meta-analytic approach, analyzing the overall strength of the association between the two variables and the possible moderators. The findings initially clarify the current controversies between the I-PACE Model and the Digital Stress Theory and provide evidence to support the further advancement of the topic.

It is also found a significant positive correlation between FoMO and mobile phone addiction, showing that fear of missing out may be an important factor to trigger mobile phone addiction. Psychological services and mental health services should be developed to reduce the emergence of fear of missing out in the digital age and thus alleviate dependence on devices. In addition, this study also found that the strength of the association between fear of missing out and mobile phone addiction was moderated by the measurement applied to quantify the variables. Future researchers should pay attention to the choice of instruments and find the best-suited scale with moderate questions and high reliability for cell phone addiction to quantify it more accurately.

This study has some limitations. Firstly, this study only focuses on the characteristic variables that affect the relationship between fear of missing out and mobile phone addiction. After the data is enriched, we can further analyze the psychological variables (such as personality and social comparison tendency) that may modulate the relationship between the two. Secondly, this study only examined the relationship between fear of missing out and general mobile phone addiction. Future studies should focus on the relationship between the fear of missing out and specific social network addictions and online game addictions. Beyond that, the cross-sectional design of the original study makes it difficult to reveal the causal relationship between fear of missing out and mobile phone addiction. Follow-up studies could apply the longitude method for further verification.

## Conclusions

(a)There is a high correlation between the fear of missing out and mobile phone addiction. The higher the fear of missing out, the higher the level of mobile phone addiction. (b) Measurement of mobile phone dependency will affect the relationship between the two variables. (c) Individual factors (gender and age), environmental factors (cultural background and publication year), and measurement of FoMO had no significant effect on the correlation between fear of missing out and mobile phone addiction.

## Data Availability

The datasets used and analysed in this study are available online.
